# Comparison of the Effectiveness of Colostrum, Colostrum Powder and Combined Feeding on Transfer of Passive Immunity in Calves

**DOI:** 10.1002/vms3.71106

**Published:** 2026-07-20

**Authors:** Yiğit Kaçar, Mehmet Emin Akkaş

**Affiliations:** ^1^ Bursa Uludag University Faculty of Veterinary Medicine, Department of Internal Medicine Bursa Türkiye

**Keywords:** Brix%, failed transfer of passive immunity, Holstein‐Friesian, immunoglobulin, total protein

## Abstract

**Background:**

High‐quality colostrum is essential to prevent failed transfer of passive immunity (FTPI) in calves, and research on colostrum substitutes has continued over time when quality colostrum is unavailable.

**Objectives:**

This study evaluated the effects of fresh colostrum (COL), colostrum powder (POW) and a combination (MIX) on TPI in neonatal calves.

**Methods:**

Forty‐five neonatal Holstein‐Friesian calves were divided into COL (4 L fresh colostrum), POW (1080 g colostrum powder in 4 L water) and MIX (540 g powder in 4 L colostrum) groups. Total protein (TP), Brix%, and IgG concentrations were measured at 0 and 24 h. Receiver operating characteristic (ROC) analysis and logistic regression assessed their predictive power for FTPI.

**Results:**

At 24 h, the Brix%, TP and IgG values for the COL, POW and MIX groups were as follows (mean ± standard error; median [interquartile range]): Brix%: 9.4 ± 0.3 (9.4 [8.2–10]), 9.5 ± 0.2 (9.6 [8.8–9.8]) and 10.2 ± 0.2 (10.4 [9.6–10.8]); TP(g/dL): 5.9 ± 0.2 (5.8 [4.8–6.8]), 5.9 ± 0.2 (5.8 [5.5–6.2]) and 6.7 ± 0.2 (6.6 [6.2–7.4]); IgG(g/L): 16.09 ± 0.48 (16.31 [15.02–17.8]), 15.02 ± 0.58 (14.91 [13.05–17.68]) and 18.03 ± 0.29 (18.04 [16.91–19.25]), respectively. Compared to the POW and COL groups, the MIX group had higher IgG, TP and Brix% values at 24 h. Area under the curve (AUC) values were 0.72, 0.73 and 0.82 for Brix%, TP and IgG, respectively, with IgG showing the highest discriminatory power among the evaluated parameters.

**Conclusions:**

Colostrum powder was as effective as fresh colostrum in providing sufficient TPI. Combining colostrum powder with quality colostrum further enhances immunity levels.

## Graphical abstract

Serum IgG, total protein and Brix values were compared in calves fed natural colostrum, colostrum powder or a combination of both. Combined feeding improved passive transfer indicators, suggesting that colostrum powder supplementation may enhance passive immunity when high‐quality colostrum is limited.

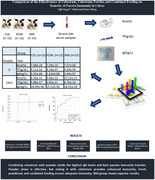

## 1 Introductıon

Calves are born agammaglobulinemic (Weaver et al. 2000; Mcguirk and Collins 2004; Armengol and Fraile 2016) or hypogammaglobulinemic (Quigley Iii et al. 2002; Topal et al. 2018) due to insufficient transfer of immunoglobulins during pregnancy, caused by the syndesmochorial placental structure in cattle. For their survival, calves must consume high‐quality colostrum immediately after birth (Weaver et al. 2000; Blum 2006; Batmaz 2015; Yang et al. 2015; Hernandez‐Castellano et al. 2014). Colostrum, produced in the mammary glands during the first 3 days postpartum (Solomons 2002; Dzik et al. 2017), contains over 90 biologically active components (Dzik et al. 2017), including immunoglobulins, vitamins, minerals, growth factors, lactoperoxidase and lactalbumin. Colostrum with these components supports the calf's nutrition and strengthens its immune system (Thapa 2005; Godden 2008; Hernandez‐Castellano et al. 2014; Kaçar and Batmaz 2023; Graydon et al. 2024).

Passive transfer is the absorption of maternal immunoglobulins from colostrum into the bloodstream of the calf within the first 24 h after birth, providing protection against common diseases until the calf's immature immune system becomes functional (Weaver et al. 2000; Blum 2006). Failure to achieve this status within 24 h for various reasons and serum IgG (IgG1) below 10 g/L is defined as failed transfer of passive immunity (FTPI) (Tyler et al. 1996; Weaver et al. 2000; Bielmann et al. 2010; Lombard et al. 2020; Lichtmannsperger et al. 2023).

FTPI is common worldwide. Several studies have investigated passive transfer in dairy calves. On the basis of data from 1816 calves aged between 1 and 7 days across 17 states, 40.7% of cattle farms in the USA reported at least one case of FTPI (Beam et al. 2009); on the other hand, a study involving 2545 pre‐weaned dairy calves from 104 farms across 13 states found FTPI in 13% of the calves (Urie et al. 2018). In Canada, FTPI was present at rates ranging from 3% to 39% in 18 farms with at least 100 lactating cows (Atkinson et al. 2017). In another study conducted in Canada, 217 calves from 30 farms were included, with a reported FTPI prevalence of 43.3% (Elsohaby et al. 2019). Prevalence rates are similar in other countries, 31%–34% in New Zealand (Cuttance et al. 2017) and 34% in Czech Republic (Staněk et al. 2019). A recent meta‐analysis from Australia and New Zealand reported an average FTPI prevalence of 33% and a farm‐level prevalence of 38% (Van et al. 2023). Similarly, Weaver et al. (2000) found that nearly one in three newborn calves experienced FTPI. In Turkey, approximately 20% of farms with advanced management systems were reported to have calves with FTPI (Kara and Ceylan 2021), whereas FTPI prevalence at the calf level ranged from 22.4% to 39% (Topal 2018; Sağlam et al. 2026).

FTPI increases calves’ susceptibility to diseases, neonatal mortality and poor weight gain, potentially reducing long‐term productivity (Sentürk 2018). In financial terms, the average loss per calf was estimated €60 on dairy farms and €80 on beef farms (Raboisson et al. 2016). Therefore, ensuring sufficient immunoglobulin intake through passive transfer is critical for calf survival and future productivity.

IgG concentrations are typically measured by ELISA or RID to determine colostrum quality, with concentrations of 50 g/L or more considered sufficient quality (Godden et al. 2019). For adequate TPI, calves should consume at least 150–200 g of IgG (Batmaz 2021a). In addition to direct IgG measurement, Brix%, total protein (TP) and gamma‐glutamyl transferase (GGT) enzyme activity provide practical semiquantitative methods and a rapid means to assess colostrum quality (Zanker et al. 2001; Quigley et al. 2013; Bartier et al. 2015; Topal et al. 2018; Batmaz 2021a; Kaçar et al. 2021). In the previous study, colostrum with a %Brix value ≥27% was categorized as high‐quality and those with a %Brix value <22% as low‐quality (Kaçar and Batmaz 2023). Moreover, it has been stated that for colostrum, a brix value of 27% is equivalent to approximately 60 g/L IgG (Batmaz 2021b).

Various studies evaluating the quality of cow colostrum showed that 12%–29% of colostrum samples were found to be of poor quality (Levieux and Ollier 1999; Bartier et al. 2015; Kara and Ceylan 2021; Kul et al. 2022). In a study conducted in Poland, Puppel et al. (2020) emphasized that approximately 60% of colostrum samples are insufficient to provide adequate immunity in calves. The selection and use of high‐quality colostrum in the prevention of FTPI is of particular importance in the Holstein breed because it is the most widely used cattle breed in dairy farms worldwide (Foksha and Konstandoglo 2019). The quality of colostrum produced by Holstein cows is lower than that of many other cattle breeds (Weaver et al. 2000; Kara and Ceylan 2021).

To prevent or mitigate the consequences of FTPI, several alternative approaches have been investigated, including hyperimmune serum preparations, immunomodulatory products, homoeopathic preparations, commercial whey‐based colostrum replacers and immunoglobulin paste formulations derived from animal or plant protein sources. These products are intended to enhance passive immunity in calves when adequate high‐quality colostrum is unavailable, although their efficacy remains a subject of debate (Swan et al. 2007; Quezada‐Tristán et al. 2014; De Paula et al. 2019; Lopez et al. 2020; Van Soest et al. 2020; Ahmadi et al. 2021; Silva et al. 2024). This study aims to assess the impact of fresh colostrum, colostrum powders and their combination on FTPI levels in calves. The cut‐off values and predictive value of other feeding methods (powder and combination) for identifying FTPI when colostrum feeding is considered the gold standard were also determined.

## 2 Materials and Methods

This study was carried out with the approval of the Bursa Uludag University Animal Experiments Local Ethics Committee (2023–09/04). The animal material of the study consisted of Holstein‐Friesian cows and 45 newborn calves of both sexes, 0–2 days old and approximately 45–50 kg body weight, born from these cows in the cattle farm (farm no: TR1600631) in Bursa/Karacabey (40° 12′ 57.7368″ North and 28° 21′ 32.3964″ East) with same care and feeding condition in the farm. Only healthy calves from cows that gave birth without complications were included in the study. Fresh colostrum used in the COL and MIX groups was obtained from the calves’ own dams immediately after parturition and fed shortly after collection without prolonged storage. Only first‐milking colostrum with a Brix value of ≥27% and free of blood, clots and purulent material was considered suitable for use; calves receiving colostrum that did not meet these criteria were excluded from the study. Calves with conditions, such as dystocia, premature birth, congenital anomalies, aspiration pneumonia, weak suckling reflexes or insufficient colostrum intake, were excluded from the study. As pregnancies on the farm were planned by artificial insemination method and delivery time was estimated by routine pregnancy examinations, cows were closely watched at the time of delivery. As a farm routine, all cows were included in dry period groups in the last 2 months of gestation and vaccinated with Rotavec Corona (MSD Animal Health, Burgwedel, Germany) and Coglavax (CEVA, Budapest, Hungary) in the dry period. It is also known that all animals are vaccinated twice a year with foot and mouth vaccine (Turkvac‐oil, Ankara, Turkey) and antiparasitic (Eprecis, CEVA, Budapest, Hungary).

In the study, calves were separated from their mothers before colostrum consumption (within the first 30 min) after birth and divided into three equal groups of 15 calves each as described below. The first colostrum feeding was administered within 2 h after birth using a nipple bottle. For calves born during the night, the first 2 L of colostrum were administered within 30 min after birth. The remaining 2 L were provided approximately 12 h later, ensuring that each calf received a total of 4 L of colostrum within the first 24 h of life.

Group COL: Calves in this group were fed with the fresh colostrum of their maternal cows after blood sampling. These calves were fed 4 L of colostrum (2 L in the morning and 2 L in the evening) on the first day according to the routine protocol of the farm. The Brix% value of colostrum was noted before the calves were given the colostrum.

Group POW: Calves in this group were fed only colostrum powder (produced by the Revolving Fund Enterprise of Uşak Provincial Directorate of Agriculture and Forestry, Turkey; registration/approval no.: YK‐TR‐6400431) on the first day after blood sampling. The calves were fed 1080 g of colostrum powder dissolved in 4 L of water (2 L in the morning and 2 L in the evening) on the first day, similar to the feeding of colostrum. Colostrum powder was reconstituted in water at approximately 40°C and administered to calves at approximately 38°C. The colostrum powders used in the study were obtained by the dry freeze technology applied to colostrum collected from disease‐free farms, and they contain 40% crude protein, 15% crude fat, 2.5% crude ash and 95% dry matter. As the Brix% value provides an estimate of dry matter content, colostrum powders were prepared by mixing 270 g of colostrum powder per litre of water. Colostrum powders used in this group include a mean IgG content of 70.5 g/L.

Group MIX: Calves in this group were fed with their maternal colostrum and powder after blood sampling. On the first day, in addition to the routine farm protocol (2 L of colostrum in the morning and 2 L in the evening), 270 g of colostrum powder was added directly to each 2‐L portion of fresh colostrum, resulting in a total of 540 g of powder administered within the first 24 h.

Calves in the COL and MIX groups were fed colostrum with a Brix% value of at least 27 (range: 27–35), with the mean IgG content of this fresh colostrum determined as 78.8 g/L. Colostrums with low Brix%, bloody, purulent and clotted colostrums were not used in the feeding of any calf. Calves in all three groups received transitional milk on Days 2 and 3, followed by whole milk from Day 4 onwards. All calves were housed in individual cages until weaning and monitored for the occurrence and duration of diarrhoea during the first month of life using a faecal scoring system (1–5), with scores of 4 and 5 classified as diarrhoea (Batmaz 2021c).

Within the study, 10 mL serum blood samples (using yellow/gel tubes) were collected from the *vena jugularis* of all calves twice, the first time within the first 30 min after birth (before colostrum/colostrum powder/colostrum and colostrum powder consumption) and the second time at 24 ± 1 h of age. Blood samples were centrifuged at 1008×*g* to obtain blood serum. Serum samples were stored at −20°C until laboratory analysis.

Brix% and TP concentrations in serum samples collected from calves were determined using a Brix refractometer (Milwaukee MA882, Hungary) and optical refractometer (Atago Sur‐Ne Clinical, Japan), respectively, at Bursa Uludag University Faculty of Veterinary Medicine Animal Hospital. IgG concentrations in calf blood serum, cow colostrum and colostrum powders were determined using a bovine IgG ELISA kit (BIO K 420‐MonoScreen QuantELISA Immunoglobulin Easy, Belgium) according to the test application instructions.

Statistical analyses were performed using SigmaPlot software (Version 15; Systat Software CA, USA) and MedCalc software (Version 19; MedCalc Software Ltd., Ostend, Belgium). Data were analysed for normality distribution (Shapiro–Wilk test) and homogeneity of variance assumptions (Brown–Forsythe test). A two‐way analysis of variance (two‐way ANOVA) was conducted to evaluate the effects of group (COL, POW and MIX) and time (0 and 24 h) on the measured parameters. The analysis was performed separately for each variable, including serum IgG concentration, Brix% and TP concentrations. When significant main effects or interactions were identified, Holm–Sidak post hoc test was applied for pairwise multiple comparisons. The correlations between parameters were evaluated with the Spearman correlation test. The percentage increases of Brix, TP and IgG concentrations between 0 and 24 h were calculated using the formula [(24‐h measurement–0‐h measurement)/0‐h measurement].

The predictive power of different feeding regimes (COL, POW and MIX) on TPI based on Brix%, TP and IgG concentrations was evaluated using receiver operating characteristic (ROC) analysis. Fresh colostrum feeding was considered the gold standard for ensuring adequate TPI in calf health, and the COL group was used as the reference in the ROC analysis. For each parameter, cut‐off values were determined using Youden's J index, and sensitivity, specificity and area under the curve (AUC) values were reported. For groups with statistically significant results, logistic regression analysis was performed using the cut‐off values. Brix%, TP and IgG concentrations were included in the model as independent variables to identify which parameter had the strongest predictive power for FTPI based on odds ratios and confidence intervals (CIs). ROC curves and dot plots were generated to illustrate the findings. In all evaluations, *p* < 0.05 was considered significant.

## 3 Results

Descriptive statistics for Brix%, TP and IgG concentrations at 0 and 24 h are summarised in Table 1. In the COL, POW and MIX groups, the percentage increases from 0 to 24 h were 21.3%, 22.3% and 25.9% for Brix% values and 30.1%, 29.6% and 34.4% for TP values, respectively. For IgG concentrations, the increases were markedly greater, corresponding to approximately 6.8‐fold (584.7%), 6.4‐fold (544.6%), and 7.4‐fold (638.9%) increases in the COL, POW and MIX groups, respectively. In the Spearman correlation analysis performed with 0 h data; *r*:0.87 (*p* < 0.01), *r*:0.63 (*p* < 0.01), *r*:0.77 (*p* < 0.01) and positive correlation were found between TP and Brix% values in COL, POW and MIX groups, respectively (*p* < 0.05). Similarly, Spearman correlation analysis using the 24‐h data revealed strong positive correlations between TP and Brix% values in all groups: COL (*r* = 1, *p* < 0.01), POW (*r* = 0.98, *p* < 0.01) and MIX (*r* = 0.93, *p* < 0.01).

**TABLE 1:** Brix%, total protein (TP) and IgG concentrations in serum of calves at 0 and 24 h.

		Group		
Time(h)/Test		COL (*n* = 15)	POW (*n* = 15)	MIX (*n* = 15)
0	Brix(%) Median(min–max)	7.38 ± 0.18 7.4 (6.4–9.1)	7.34 ± 0.10 7.4 (6.6–8)	7.57 ± 0.08 7.6 (6.9–8)
	TP(g/dL) Median(min–max)	4.11 ± 0.16 4.1 (3.4–5.6)	4.16 ± 0.07 4.1 (3.7–4.7)	4.39 ± 0.09 4.5 (3.5–4.9)
	IgG(g/L) Median(min–max)	2.35 ± 0.08 2.31 (1.96–2.93)	2.33 ± 0.08 2.13 (1.86–2.94)	2.44 ± 0.07 2.49 (1.94–2.92)
24 ± 1	Brix(%) Median(min–max)	9.38 ± 0.33 9.4 (7.6–11.8)	9.46 ± 0.23 9.6 (8.2–11.8)	10.23 ± 0.21 10.4 (9–11.4)
	TP(g/dL) Median(min–max)	5.88 ± 0.27 5.8 (4.2–7.8)	5.92 ± 0.21 5.8 (4.8–8.2)	6.70 ± 0.18 6.6 (5.6–7.6)
	IgG(g/L) Median(min–max)	16.09 ± 0.48 16.31 (12.21–18)	15.02 ± 0.58 14.91 (11.25–18.43)	18.03 ± 0.29 18.03 (15.93–19.54)

The effects of group and time on Brix%, TP, and serum IgG concentrations were evaluated using two‐way ANOVA, and the results are presented in Table 2. A significant effect of group was observed for all three variables, whereas a significant interaction effect (group × time) was detected only for IgG concentrations. Pairwise comparisons using the Holm–Sidak post hoc test are summarized in Table 3. Two‐way ANOVA revealed significant effects of treatment group and time on serum Brix%, TP, and IgG levels (all *p* < 0.01), with a significant interaction effect observed for IgG (*p* < 0.01) but not for Brix% or TP (*p* > 0.05). At 0 h, no significant differences were observed among the groups for any parameter (*p* > 0.05), indicating a comparable baseline prior to colostrum intake. Post hoc comparisons (Holm–Sidak test) demonstrated that the MIX group consistently outperformed the other treatments: Brix%: MIX values (10.23% ± 0.21%) were significantly higher than COL (9.38% ± 0.33%; *p* = 0.01), but not statistically different from POW (*p* = 0.07). TP: MIX (6.70 ± 0.18 g/dL) was significantly higher than both COL (5.88 ± 0.27 g/dL; *p* < 0.01) and POW (5.92 ± 0.21 g/dL; *p* < 0.01). IgG at 24 h: MIX (18.03 ± 0.29 g/L) showed markedly elevated levels compared to POW (15.02 ± 0.58 g/L; *p* < 0.01) and COL (16.09 ± 0.48 g/L; *p* < 0.01); COL also exceeded POW (*p* = 0.03). Time‐dependent increases were significant in all groups for Brix%, TP and IgG levels from 0 to 24 h (*p* < 0.01), reflecting the expected physiological changes following colostrum intake. The superior IgG concentrations in the MIX group at 24 h (Δ = +3.0 g/L vs. POW) highlight its enhanced efficacy in promoting passive immunity.

**TABLE 2:** Two‐way analysis of variance (two‐way ANOVA) results (Brix%, TP and IgG).

Variable	Source of variation	df	Sum of squares (SS)	Mean square (MS)	*F* value	*p* value
Brix(%)	Group	2	5.72	2.86	4.54	0.01*
	Time	1	116.96	116.96	185.61	<0.01*
	Group × time	2	0.98	0.49	0.78	0.46
TP (g/dL)	Group	2	7.09	3.55	7.60	<0.01*
	Time	1	86.83	86.83	186.07	<0.01*
	Group × time	2	0.54	0.27	0.58	0.56
IgG (g/L)	Group	2	39.58	19.79	11.70	<0.01*
	Time	1	4435.94	4435.94	2623.615	<0.01*
	Group × time	2	30.48	15.24	9.01	<0.01*

Abbreviation: df, degrees of freedom.

*Statistically significant (*p* < 0.05), group × time: interaction effect (significant for IgG only).

**TABLE 3:** Holm‐Sidak post hoc comparisons.

Variable	Comparison	Mean difference	*t* value	*p* value	Significant (*p* < 0.05)
**Brix(%)**	MIX vs. COL	0.6	2.91	0.01^*^	Yes
	MIX vs. POW	0.44	2.13	0.07	No
	POW vs. COL	0.16	0.78	0.43	No
**TP (g/dL)**	MIX vs. COL	0.62	3.53	<0.01^*^	Yes
	MIX vs. POW	0.56	3.19	<0.01^*^	Yes
	POW vs. COL	0.06	0.34	0.73	No
**IgG (g/L) (24 h)**	MIX vs. POW	3.01	6.33	<0.01^*^	Yes
	MIX vs. COL	1.94	4.08	<0.01^*^	Yes
	COL vs. POW	1.07	2.25	0.03^*^	Yes
**IgG (g/L) (0 h)**	All comparisons	NS (*p* > 0.05)			No

* Statistically significant (*p* < 0.05).

**FIGURE 1:**
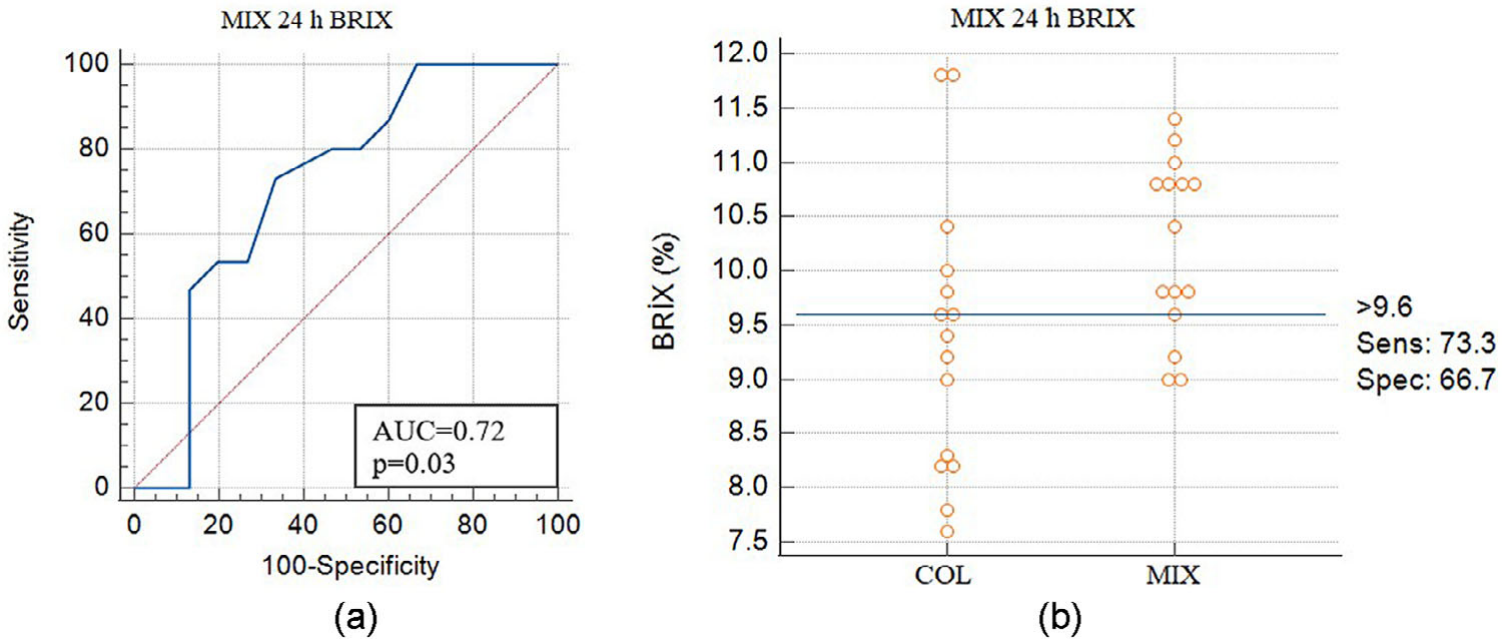
(a) ROC curve of Brix values at 24 h for MIX and COL groups. (b) Dot plot graph of Brix values at 24 h for MIX and COL groups.

**FIGURE 2:**
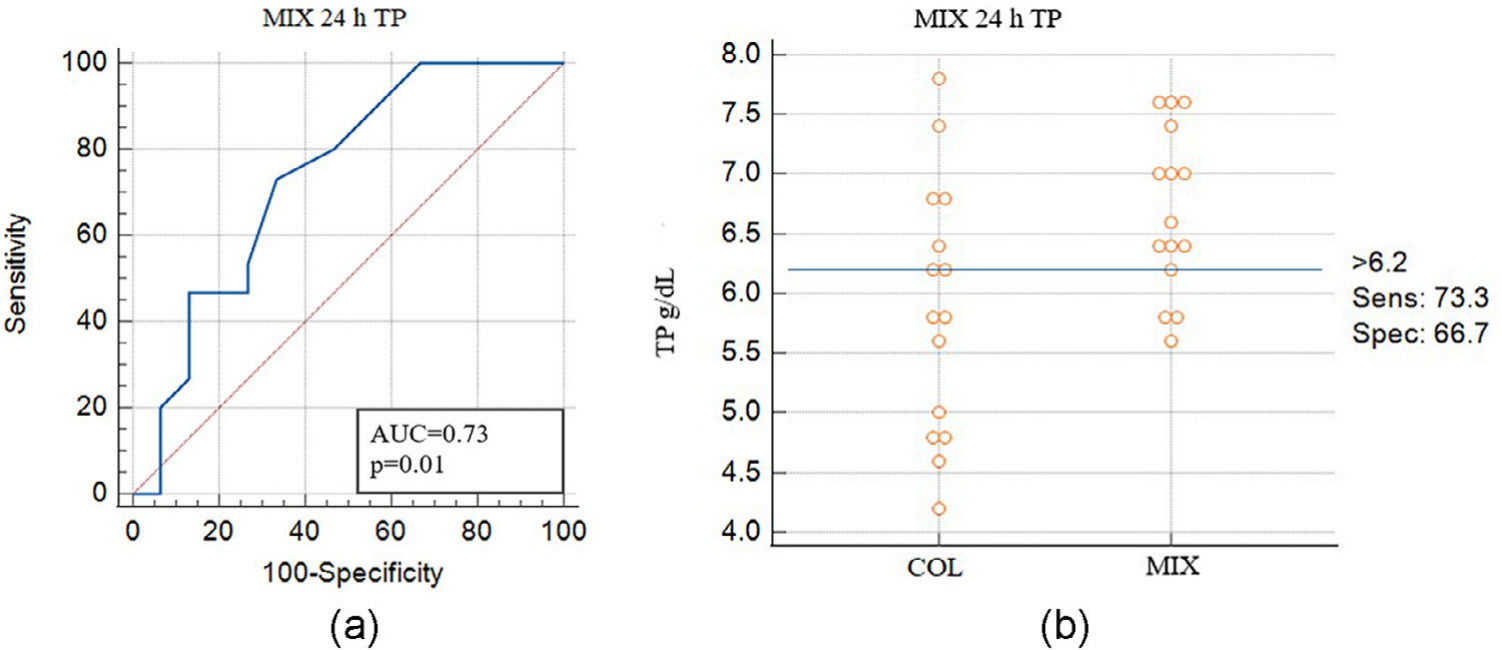
(a) ROC curve of TP values at 24 h for MIX and COL groups. (b) Dot plot graph of TP values at 24 h for MIX and COL groups. TP, total protein.

**FIGURE 3:**
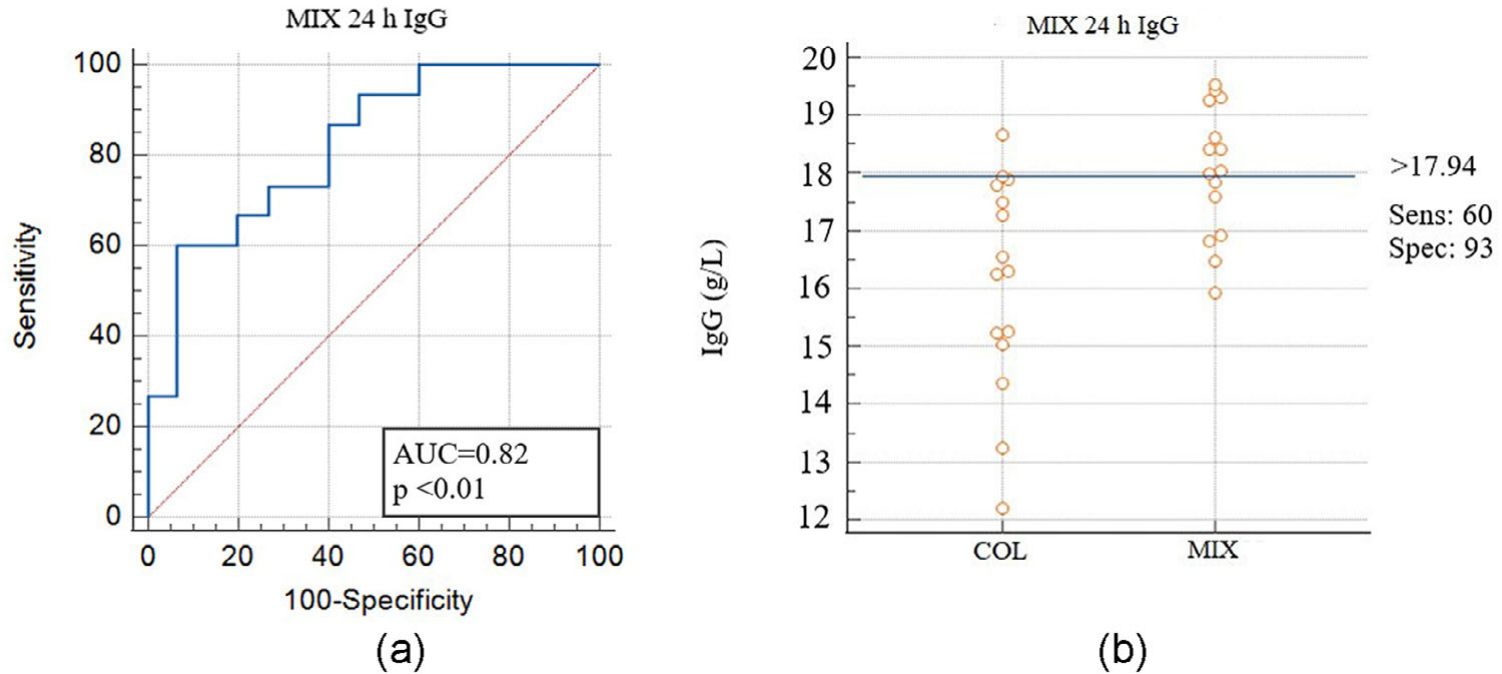
(a) ROC curve of IgG values at 24 h for MIX and COL groups. (b) Dot plot graph of IgG values at 24 h for MIX and COL groups. AUC, area under the curve.

The ROC analysis was carried out for the cut‐off values, sensitivity, specificity, Youden's J index, AUC, standard error and 95% CI on the basis of the fresh colostrum group (COL) as the gold standard. ROC analyses were performed for all groups but were indicated which had significant importance according to *p* < 0.05 (Table 4). Logistic regression analysis was conducted for the groups identified as significant in the ROC analysis. The odds ratio for IgG concentrations in the MIX group was determined to be 1.01 (*p* < 0.01). When the 24‐h data of the MIX group and the COL group were compared, the ROC curve and dot plots related to Brix% are presented in Figure 1a,b, respectively; the ROC and dot plots based on TP values are shown in Figure 2a,b; and the results for IgG values are displayed in Figure 3a,b.

**TABLE 4:** The logistic regression analysis results of the values that showed a significant difference in the receiver operating characteristic (ROC) analysis compared to the COL group.

	Youden's J	95% CI for AUC	Sensitivity	Specificity	*p* value	Cut‐off	AUC	Log Reg	
								Odds ratio	*p* value
24_Brix_MIX	0.40	0.52–0.87	73.3	66.7	0.03	>9.6	0.72	0.90	0.92
24_TP_MIX	0.40	0.54–0.88	73.3	66.7	0.01	>6.2	0.73	3.12	0.08
24_ıgG_MIX	0.53	0.64–0.94	60.0	93.0	<0.01	>17.94	0.82	1.01	0.02

*Note*: ROC analysis were performed for all groups but were indicated in which had significantly importance according to *p* < 0.05.

Abbreviations: AUC, area under the curve; CI, confidence interval.

On the basis of previously established cut‐off values (<5.5 g/dL for serum TP and <8.5% for Brix%), five calves in the COL group and three calves in the POW group fell below one or both of these indirect field‐based thresholds, whereas no calves in the MIX group fell below these thresholds.

On the basis of faecal scores, diarrhoea was observed in eight calves each in the COL and POW groups, and in five calves in the MIX group. Among affected calves, the mean (±SD) age at onset of diarrhoea was 6.13 ± 2.80, 5.88 ± 2.42 and 4.60 ± 0.89 days in the COL, POW and MIX groups, respectively. The mean (±SD) duration of diarrhoea in these affected calves was 1.93 ± 2.43, 2.00 ± 2.33 and 0.80 ± 1.42 days, respectively. Despite no etiological analysis being performed to identify the causative agents, these values are presented for descriptive purposes only due to the limited number of affected calves per group, and no statistical comparisons were performed.

## 4 Discussion

In studies evaluating colostrum quality in cattle, it is known that colostrums containing at least 60 g/L IgG are of high quality, and these colostrums have a Brix% value of at least 27% (Bartens et al. 2016; Batmaz 2016; Batmaz 2021b). In this study, fresh colostrums with a Brix% value of at least 27% and suspensions prepared by adding 270‐g packages of colostrum powders obtained from quality colostrums with dry freeze technology to 1 L were used. Thus, it is seen that the study was designed to ensure that all groups received quality colostrum.

IgG measurement is the gold standard in the evaluation of passive transfer levels in calves (Tyler et al. 1996; Weaver et al. 2000; Bielmann et al. 2010). The study demonstrated that the serum IgG concentrations before the first feeding (colostrum/colostrum powder or a mixture) were low and similar across all three groups, indicating that the groups were created under the same conditions. Furthermore, these IgG concentrations were consistent with previous studies, confirming that the first samples from the calves were indeed taken before the first feeding (Holloway et al. 2002; Topal et al. 2018). When 24th‐h IgG concentrations were evaluated in all groups, no FTPI was found in any group. At 24 h, the MIX group exhibited statistically higher IgG concentrations (*p* < 0.05) (Table 3). Similarly, Zaremba et al. (1993), in their study evaluating the effects of different feeding methods on calf TPI levels, found the IgG concentrations to be highest to lowest in the following order: Combination, fresh colostrum and only colostrum powder. In addition, the mean IgG value of the calves in the COL group at 24 h was significantly higher than that of the POW group (*p* = 0.03), which is in line with previous studies (Zaremba et al. 1993; Garry et al. 1996; Holloway et al. 2002). Previous studies comparing natural colostrum with commercial replacers have reported lower serum IgG concentrations and higher FTPI rates in calves fed replacers (Swan et al. 2007; Ahmadi et al. 2021). The absence of FPTI in all groups can be explained by the fact that the colostrum powders had a Brix value of 27%, and the fresh colostrum administered to calves in the COL group was intentionally selected to have a Brix% of at least 27, thereby ensuring sufficient IgG content for effective passive transfer. Furthermore, it is anticipated that using colostrum powders obtained through ‘dry freeze’ technology in the presented study has contributed to effective TPI by reducing IgG loss in the powder production process.

Except for IgG values, serum Brix% and TP values are often used practically to determine the level of passive transfer in calves under farm conditions (Topal et al. 2018). Considering the commonly used serum Brix% and TP thresholds reported in previous studies (Topal et al. 2018), five calves in the COL group and three calves in the POW group fell below the cut‐off values of <5.5 g/dL for serum TP concentration (Weaver et al. 2000) and <8.5% for serum Brix% (Hernandez et al. 2016), whereas no calves in the MIX group fell below these thresholds. This apparent discrepancy is not unexpected because serum IgG concentration is considered the reference method for evaluating passive transfer, whereas serum TP and Brix measurements are indirect field‐based indicators. Therefore, complete agreement among these methods is not always expected. This can be explained by the fact that the 24‐h serum IgG, TP and Brix% values observed in calves from the MIX group were significantly (*p* < 0.05) higher than those in the other two groups. In line with previous literature (Topal et al. 2018), the differences observed between IgG‐based and indirect field‐based classifications in the present study further support the limited sensitivity of serum TP and Brix% measurements for identifying calves with inadequate passive transfer. In this research, the ROC analysis indicated that IgG concentrations in the MIX group demonstrated the highest diagnostic performance, with an AUC of 0.82 (95% CI: 0.64–0.94), the highest specificity (93%) and a sensitivity of 60%. Logistic regression analysis also identified IgG concentration as a significant predictor (OR = 1.01, *p* = 0.02). Additionally, Brix% and TP concentrations showed moderate diagnostic performance with AUC values of 0.72 and 0.73, respectively, supporting their potential as complementary diagnostic tools. Similarly, the strong correlations observed between Brix% and TP concentrations in this study are consistent with previous findings (Hernandez et al. 2016), suggesting that these tests may serve as practical field‐based screening tools for assessing passive transfer status in calves.

The numerically lower incidence and shorter duration of diarrhoea observed in the group receiving colostrum supplementation are in agreement with previous studies (Berge et al. 2009; Lago et al. 2026). For example, Berge et al. (2009) reported that the addition of colostrum‐derived immunoglobulin supplements to milk replacer during the first 2 weeks of life reduced both the duration of diarrhoea episodes and the need for antimicrobial treatment in neonatal calves. Similarly, Lago et al. (2026) demonstrated that supplementation of milk replacer with a whey‐based colostrum replacer during the first 14 days of life reduced disease treatments and mortality while improving early growth performance in calves. The observations in our study are generally consistent with these previous findings. However, because diarrhoea outcomes were recorded for descriptive purposes only and no etiological investigations were performed, the potential effects of colostrum supplementation on calf health and diarrhoea outcomes should be interpreted with caution. Furthermore, Schinwald et al. (2022) demonstrated that prolonged diarrhoea episodes were negatively correlated with daily live weight gain in Holstein male calves, highlighting that diarrhoea not only impairs calf health but can also adversely affect growth performance and farm profitability. These findings collectively highlight the potential importance of effective nutritional strategies in the early life of calves.

This study has some limitations that should be considered when interpreting the findings. First, the number of calves included in each group was relatively small, which may have limited the statistical power to detect differences in some outcome variables. Second, the study was conducted on a single commercial dairy farm under specific management and feeding conditions. Therefore, the results should be interpreted with caution and may not be directly generalizable to herds with different management practices, colostrum‐feeding protocols, environmental conditions or health status. Further studies involving larger numbers of calves from multiple farms are needed to confirm the present findings and improve their broader applicability.

As a conclusion, the study revealed that colostrum and colostrum powder produced similar results in terms of passive transfer of immunity, as well as the number of diarrhoea cases and diarrhoea days. It was also determined that colostrum powder given in addition to quality colostrum was effective in improving passive transfer levels and reducing the number of days with diarrhoea. These findings suggest that colostrum powder can be considered a practical alternative feeding strategy under farm conditions where adequate amounts of high‐quality colostrum are not available, such as when colostrum supply is insufficient, colostrum quality is inadequate, or when calves are born to heifers or cows that cannot provide enough colostrum. Future studies should investigate the effectiveness of colostrum powder under different farm conditions and examine its long‐term effects on calf health and productivity.

## Author Contributions

**Yiğit Kaçar**: conceptualization, methodology, data curation, investigation, validation, formal analysis, supervision, funding acquisition, visualization, project administration, resources, writing – original draft, and writing – review and editing. **Mehmet Emin Akkaş**: methodology, data curation, investigation, formal analysis, visualization, writing – original draft, and writing – review and editing.

## Funding

The authors have nothing to report.

## Ethics Statement

This study was carried out with the approval of the Bursa Uludag University Animal Experiments Local Ethics Committee (2023–09/04). The authors confirm that the ethical policies of the journal, as noted on the journal's author guidelines page, have been adhered to and the appropriate ethical review committee approval has been received.

## Conflicts of Interest

The authors declare no conflicts of interest.

## Data Availability

The data that support the findings of this study are available from the corresponding author upon reasonable request.
